# The incidence of cross infections in Imam Reza hospital, Mashhad, Iran

**Published:** 2012-12

**Authors:** A Javanbakht, E Askari, L Danesh, N Moghadas, I Mostafavi, M NaderiNasab

**Affiliations:** 1 Mashhad Medical Microbiology Student Research Group, Mashhad University of Medical Sciences, Mashhad, Iran; 2 Committee of Infection Control, Imam Reza Hospital, Mashhad University of Medical Sciences, Mashhad, Iran; 3 Microbiology and Virology Research Center, Avicenna Research Institute; 4 Department of Medical Bacteriology & Virology, Emam Reza Hospital, Faculty of Medicine, Mashhad University of Medical Sciences, Mashhad, Iran

**Keywords:** Cross Infections, *Acinetobacter*, *Iran*

## Abstract

**Background and Objectives:**

Cross Infections (CIs) are considered as a major public health problem worldwide. They cause delay in recovery, increased mortality and morbidity. The purpose of this study is to report the incidence of CIs in our teaching hospital during a 23-month period.

**Materials and Methods:**

In this descriptive cross-sectional study, 76766 patients from 2009 to 2011 admitted to 17 wards of Imam Reza Hospital in Mashhad were studied for CIs. Patients’ age, sex, site of infection, ward of hospitalization and type of microbial infections were collected and analyzed by SPSS 16.0.

**Conclusion:**

Results of the present study showed that the incidence of CIs was low (i.e.<1%) in our hospital and *Acinetobacter* (25.8%, n = 176) was the most frequent pathogen.

## INTRODUCTION

Cross Infections (CIs) are infections that occur after 48 hours of hospitalization in uninfected patients at admission (1). Inappropriate antimicrobial treatment of CIs is associated with delay in recovery, increased mortality, morbidity and length of stay in hospitals. In developing countries, lack of staff and resources are considered the most reason for not well established prevalence of CIs.

The purpose of this study is to report the incidence of CIs in a teaching hospital in Mashhad, Iran during a 23-month period.

## MATERIALS AND METHODS

In this descriptive cross-sectional study, from January 2009 to December 2011, patients admitted to Imam Reza Hospital in Mashhad were studied for CIs. We obtained the patients information from hospital information system with the help of the members in infection control committee. CIs were categorized according to National Nosocomial Infection Surveillance (NNIS) system (2).

Surgical site infections (SSIs) were defined as infections occurring at least 2 days after the operative procedure and involving the site of incision, with at least 1 of the following: purulent discharge from the site of incision; or diagnosis of infection by the surgeon. A case of urinary tract infections (UTI) was defined as a patient with the following signs or symptoms with no other recognizable cause: fever (temperature >38°C), dysuria, or suprapubic tenderness; and positive dipstick for leukocyte esterase and/or nitrate, physician diagnosis of a UTI, or both. A case of pneumonia was defined as a patient who had rales or dullness to percussion on physical examination of the chest; abnormal chest radiography; or new onset of purulent sputum. A case of bloodstream infection (BSI) was defined as a patient with at least 1 of the following signs or symptoms with no other recognized cause: fever (temperature >38°C), hypotension (systolic pressure ≤90 mm Hg), or oliguria (<20 cm^3^/h); and no apparent infection at another site and physician instituted treatment for sepsis.

Patient's age, sex, site of infection, ward of hospitalization and type of microbial infection were analyzed by SPSS 16.0. We used chi-square test for analyzing our data. *P* < .05 was considered significant for statistical test.

Urine, respiratory and wound samples were cultured on blood and Eosin-methylene blue agar. Also, mannitol salt agar was used for isolation of staphylococci from wound samples. Gram staining of samples was also undertaken. If no growth was identified after 48 hours incubation, the sample was reported as negative. For positive samples, biochemical tests were done in order to biotype each bacterial strain. Blood cultures were done after taking 10ml blood samples from patients using bottles filled with brain heart infusion broth.

## RESULTS

Out of 76766 patients of 17 wards, 777 CIs were identified in 720 patients (50.2% female, age 43.2±24.3 years). Among 777 cultures that was requested, 681 of them were correctly identified (Table 1), 43 requests were culture negative, 53 were inaccurate diagnosis (Gram positive bacilli, Gram positive cocci and fungi).

**Table 1:** Microorganisms isolated from 681 medium according to the site of infection.

Microorganism	Urinary tract	Bloodstream	Surgical site	Pneumonia	Other sites*	Total
***Klebsiella spp. No. (%)***	25 (25.5%)	16 (16.3%)	38 (38.8%)	19 (19.4%)	0	98
***Pseudomonas aeruginosa No**. * **(%)**	5 (3.9%)	6 (4.6%)	98 (76%)	16 (12.4%)	4 (3.1%)	129
***Acinetobacter spp. No**. * **(%)**	7 (4%)	17 (9.7%)	142 (80.7%)	9 (5%)	1 (0.6%)	176
***Escherichia coli No**. * **(%)**	37 (67.3%)	3 (5.4%)	12 (21.9%)	2(3.6%)	1 (1.8%)	55
***Candida spp. No. (%)***	75 (88.2%)	2 (2.4%)	3 (3.5%)	4 (4.7%)	1 (1.2%)	85
***Staphylococcus epidermidis No**. * **(%)**	1 (25%)	2 (50%)	0	1 (25%)	0	4
***Staphylococcus aureus No**. * **(%)**	27 (20.1%)	24 (17.9%)	69 (51.6%)	7 (5.2%)	7 (5.2%)	134
**Total**	177	70	362	58	14	681

* others: peritoneal fluid and intubation tube

Totally, the incidence of CIs was 0.94% in Imam Reza Hospital in Mashhad. The highest frequency of CIs was in burn ward (34.7%, n = 327), which had significant difference compared to other wards (*P*<0.001).

SSIs were the most frequent category of infection (46.5%) followed by UTIs (22.7%), BSIs (9%) and other sites in our hospital (Table 1).

The predominant bacteria in kidney transplantation, cardiac surgery ICU, accident, emergency surgery, general surgery, accidental surgery, cardiac surgery and vascular surgery wards were *Klebsiella*. In burn ward *Acinetobacter* was the most frequent pathogen (n = 107). Totally, the most isolated bacteria in Imam Reza hospital was *Acinetobacter* (n = 176, 22.6%) followed by *Staphylococcus aureus* (*S. aureus*) (N = 134, 17.2%) and *Pseudomonas aeruginosa (P. aeruginosa)* (N = 129, 16.6%). Also *Staphylococcus epidermidis (S. epidermidis)* had the least prevalence (0.5%). (Table 1)

Among 720 patients who diagnosed to have CIs, 28.8% (N = 221) died in hospital (53.4% female, mean age 46.4±24.36 years). Among these patients 22.6% (N = 50) had *Acinetobacter* followed by *P. aeruginosa* 20.8% (N = 46) and *Candida spp* 14.5% *(*N = 32*)*. The most frequent categories of infection in these patients were the SSIs (42.5%) followed by UTIs (27.6%), and BSIs (15.8%).

## DISCUSSION

Our findings indicate that incidence of CIs in this teaching hospital decreased slightly compared to 2008 which was 0.96%. Gender was not a risk factor for CIs in our study, but in a study conducted by Oncul *et al*., males (63.9%) were more susceptible to CIs than female patients (3), although gender may not be a risk or protective factor in CIs worldwide.

We showed that the SSIs were the most common site of infection in Imam Reza hospital which is similar to results reported by Appelgren (4). In Pellizzer's study in Italy, urinary tract (28.4%), surgical sites (20.3%) and bloodstream (19.3%) were the most frequent sites of infection respectively (5). These differences may be due to the number of patients, place of study and genetic susceptibility.

Most of the CIs in this study happened in burn unit (0.34%) but in Balkhy's research, they happened in the intensive care units (ICUs) (46.7%), followed by the surgical ward (13.3%) (6). The reasons of high incidence of CIs in our burn ward are as follows: insufficient number of nurses although this ward is one of the largest and most crowded wards in the East of Iran, high sensitivity to infections among burned patients due to the failure of the skin barrier, the weakened function of neutrophils, humoral and cellular defense mechanisms result in exudates which are excellent media for microorganisms to grow (7, 8).

Our data demonstrated that Gram-negative bacteria (mainly *Acinetobacter* and *P. aeruginosa*) play a major role in causing CIs in recent years which is consistent with some other studies conducted in Iran. Sohrabi showed that *Escherichia coli* (64.3%), coagulase negative Staphylococci (11.2%) and *Klebsiella* (8.1%) were the most common microorganisms in their hospital (9). The reasons of these differences are still unknown but the high incidence of *Acinetobacter* in our study may be due to dry soil in Mashhad which is the origin of *Acinetobacter*.

Unlike previous studies (10, 11), coagulase negative staphylococci such as *S. epidermidis* showed the lowest frequency in our study. This may be due to neglecting this organism and considering it as a commensal by our healthcare workers when reporting CIs.

In conclusion, although the incidence rate of CIs in this hospital was low, it is necessary to maintain continuous surveillance and implement preventive measures by wearing sterile gloves, washing hands, using air conditioner in each patient's room, keeping infected patients away from other patients, appropriate food, and sufficient number of nurses to reduce CIs and their adverse effects.
